# Inhibition of COX2 enhances the chemosensitivity of dichloroacetate in cervical cancer cells

**DOI:** 10.18632/oncotarget.18518

**Published:** 2017-06-16

**Authors:** Bo Li, Xinzhe Li, Haojun Xiong, Peng Zhou, Zhenhong Ni, Teng Yang, Yan Zhang, Yijun Zeng, Jintao He, Fan Yang, Nan Zhang, Yuting Wang, Yingru Zheng, Fengtian He

**Affiliations:** ^1^ Department of Biochemistry and Molecular Biology, College of Basic Medical Sciences, Third Military Medical University, Chongqing 400038, China; ^2^ Battalion 17 of Students, College of Preventive Medicine, Third Military Medical University, Chongqing 400038, China; ^3^ Department of Obstetrics and Gynecology, Daping Hospital and Research Institute of Surgery, Third Military Medical University, Chongqing 400042, China

**Keywords:** dichloroacetate, COX2, celecoxib, QKI, cervical cancer

## Abstract

Dichloroacetate (DCA), a traditional mitochondria-targeting agent, has shown promising prospect as a sensitizer in fighting against malignancies including cervical cancer. But it is unclear about the effect of DCA alone on cervical tumor. Moreover, previous reports have demonstrated that the increased cyclooxygenase-2 (COX2) expression is associated with chemoresistance and poor prognosis of cervical cancer. However, it is still unknown whether COX2 can affect the sensitivity of DCA in cervical cancer cells. In this study, we found that cervical cancer cells were insensitive to DCA. Furthermore, we for the first time revealed that DCA could upregulate COX2 which impeded the chemosensitivity of DCA in cervical cancer cells. Mechanistic study showed that DCA reduced the level of RNA binding protein quaking (QKI), leading to the decay suppression of COX2 mRNA and the subsequent elevation of COX2 protein. Inhibition of COX2 using celecoxib could sensitize DCA in repressing the growth of cervical cancer cells both *in vitro* and *in vivo*. These results indicate that COX2 is a novel resistance factor of DCA, and combination of celecoxib with DCA may be beneficial to the treatment of cervical cancer.

## INTRODUCTION

Worldwide, cervical cancer is the second most common malignancy of women and is a major cause of morbidity and mortality [1]. At present, platinum and taxol-based chemotherapies are still standard paradigms in addition to surgery, but their side effects are severe and the chemoresistance has also emerged [2–5]. Therefore, it is urgent to explore novel strategies as alternatives of traditional chemotherapy. There are growing evidences that the unique metabolism is a new essential target of most solid tumors. Targeting key metabolic pathways can significantly kill numerous cancer cells including cervical cancer cells [6–7]. Among various metabolic drugs, dichloroacetate (DCA) has shown charming prospect because of its positive function in cancer therapy.

DCA, a mitochondria-targeting small molecule, has been recently demonstrated as a promising nontoxic antineoplastic agent that promotes apoptosis of cancer cells [8–10]. It acts as an inhibitor of pyruvate dehydrogenase kinase (PDK) and subsequently increases the activity of pyruvate dehydrogenase (PDH), which accelerates the flux of carbohydrates into mitochondria and thereby enhances aerobic oxidation of glucose [11]. This effect represses the growth of many kinds of tumors including non-small cell lung, metastatic breast, colon, prostate, endometrial and ovarian cancers and neuroblastoma [9–10, 12–16]. Importantly, previous study has confirmed that DCA can synergistically with cisplatin to inhibit the growth of HeLa cells [17].

Cyclooxygenase2 (COX2) is one of two COX subtypes which are the key enzymes of arachidonic acid metabolism [18]. COX enzymes catalyze arachidonic acid into prostaglandins which are important mediators of many physiological and pathophysiological processes including gastric and kidney function, and inflammation, fever and pain [19–21]. Unlike COX1, COX2 doesn't express at the basal condition but can be induced by a variety of stimuli including cytokines, oncogenes, growth factors and hormones [20–21]. It has been reported that COX2 is upregulated in different cancers and its elevation results in a poor prognosis such as axillary node and bone metastases, and chemotherapy resistance [22–26]. Inhibition of COX2 can act in a concerted way with improved therapeutic potential in invasive breast cancer, non small cell lung cancer, bladder cancer and cervical cancer [18, 27–29].

Celecoxib, a sulfonamide selective COX2 inhibitor (COXib), has been primarily used as an anti-inflammatory drug [30–33]. In recent years, celecoxib has shown charming prospects as an antitumor drug due to its anti-proliferative activity. For example, celecoxib suppresses the proliferation and survival of chronic myelogeous leukemia (CML) cells [34]. Moreover, celecoxib can also be used as a sensitizer with other antitumor drugs in the therapy of renal cell carcinoma and melanoma [35–36].

In this study, we demonstrated that DCA could induce apoptosis in cervical cancer cells, while it upregulated COX2 which resulted in the insensitivity of cervical cancer cells to DCA. Celecoxib could sensitize DCA via dramatically attenuating DCA-induced COX2. Moreover, DCA elevated COX2 through decreasing the decay of COX2 mRNA by repressing QKI. The *in vivo* experiments in nude mice showed that inhibition of COX2 with celecoxib could sensitize DCA in suppressing the growth of cervical cancer xenografts. In summary, these results indicate that COX2 is a novel resistance factor of DCA, and inhibition of COX2 may provide a potential therapeutic target for the treatment of cervical cancer.

## RESULTS

### DCA suppresses the survival of cervical cancer cells while upregulates COX2

**Figure 1:**
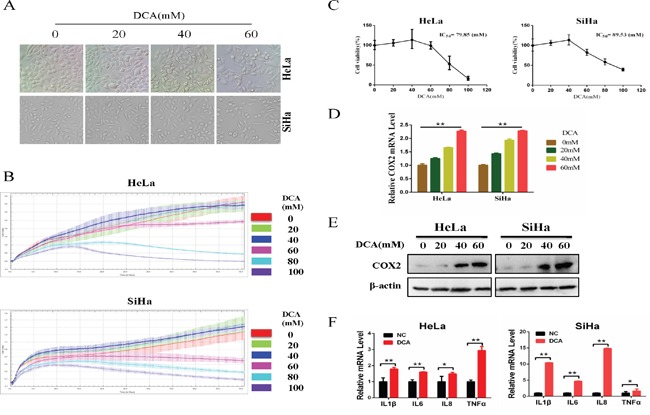
DCA suppresses the survival of cervical cancer cells while upregulates COX2 **(A)** HeLa and SiHa cells were treated with the indicated concentrations of DCA for 24 h, and then the cells were imaged under a phase-contrast microscope at 200× magnification. **(B)** HeLa and SiHa cells were treated with the indicated concentrations of DCA and the impendence of each well was recorded in a 15-min interval for 96 h using ACER xCELLigence System, and the kinetic curve of cell growth was plotted. **(C)** HeLa and SiHa cells were treated as in (A), and then the CCK8 assay was performed to evaluate the cytotoxicity of DCA. **(D-F)** HeLa and SiHa cells were treated as in (A), the mRNA level of COX2 was examined by qPCR (D), the protein level of COX2 was examined by Western blot (E) and the inflammatory factors (IL1β, IL6, IL8 and TNFα) were assessed by qPCR (F). *p<0.05; **p<0.01.

Firstly, we detected the cytotoxicity effect of DCA in HeLa and SiHa cells. As shown in Figure 1A, 60mM DCA slightly increased the natant cells compared to the control group, while 40mM DCA had little effect on cell morphology. Moreover, the results from real time cell electronic sensing (RT-CES) analysis showed that DCA dose-dependently increased the cytotoxicity effect in cervical cancer cells (Figure 1B). Nevertheless, the IC_50_ (half maximal inhibitory concentration) values of DCA in HeLa and SiHa cells were 79.85mM and 89.53mM, respectively (Figure 1C), indicating that DCA can suppress the growth of cervical cancer cells only at a high concentration. Additionally, the IC_50_ values of DCA in L02 (human normal hepatic cell) and HaCaT (immortalized human keratinocyte cell) cells were 99.93mM and 97.75mM, respectively (Supplementary Figure 1). As COX2 has been reported to be upregulated in various cancers and plays an important role in resisting cell death, we further investigated the expression of COX2 in cancer tissues using bioinformatics analysis. Results from MERAV (Metabolic gEne RApid Visualizer) indicated that the level of COX2 was elevated in FRS (female reproductive system), colon, kidney, liver and stomach tumor tissues (Supplementary Figure 2A and 2B). Furthermore, according to an analysis of the *TCGA_CESC_exp_HiSeqV2-2015-02-24* dataset, the COX2-high group had a poorer OS (overall survival) (HR=1.512, P=0.0483) than the COX2-low group (Supplementary Figure 2C). Next, to explore whether DCA could induce COX2 in cervical cancer cells, we detected the expression of COX2. As shown in Figure 1D and 1E, DCA dose-dependently increased the levels of COX2 mRNA and protein. Interestingly, DCA had no obvious effect on the expression of COX1 (another subtype of COX) (Supplementary Figure 3A and 3B), and silencing COX1 with siRNA did not enhance the chemosensitivity of DCA (Supplementary Figure 4D). In addition, the levels of 4 inflammatory factors (IL1β, IL6, IL8 and TNFα, those are downstream molecules of COX2) were also upregulated by DCA (Figure 1F). Taken together, these findings suggest that although DCA possesses an antitumor role in cervical cancer cells, the upregulated COX2 may hinder its tumor killing effect.

### Inhibition of COX2 sensitizes DCA to kill cervical cancer cells

**Figure 2:**
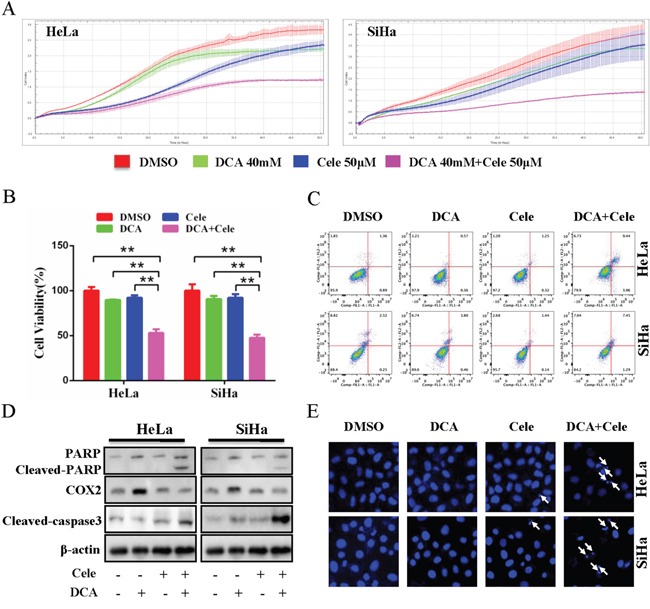
Inhibition of COX2 sensitizes DCA to kill cervical cancer cells **(A)** HeLa and SiHa cells were cotreated with 40 mM DCA and 50 mM celecoxib or each alone, and the impendence of each well was recorded in a 15-min interval for 96 h using ACER xCELLigence System, and the kinetic curve of cell growth was plotted. **(B, C)** HeLa and SiHa cells were treated as in (A) for 24 h, then the cell viability was measured by CCK8 assay (B), and the percentage of apoptotic cells was calculated using flow cytometry after stained with annexin V-FITC/PI (C). **(D)** HeLa and SiHa cells were treated as in (B), then the cleavage of PARP, cleaved caspase3 and COX2 were evaluated by Western blot. **(E)** After treated as in (B), the nucleus of HeLa and SiHa cells were stained with Hoechst 33258 and then observed under fluorescence microscope. The representative images were shown and the typical apoptotic bodies were marked with white arrows. **p<0.01.

To clarify the role of DCA-induced COX2 in the insensitivity of cervical cancer cells, HeLa and SiHa cells were treated with DCA in the presence or absence of celecoxib or siRNA against COX2 (siCOX2). As shown in Figure 2A and 2B and Supplementary Figure 4A, cotreatment with celecoxib (or siCOX2) and DCA dramatically repressed the growth of cervical cancer cells compared to DCA alone. Moreover, celecoxib enhanced the apoptosis of cervical cancer cells in response to DCA, which was revealed by Flow Cytometry analysis of annexin V-FITC (fluorescein isothiocyanate) and PI (prodium iodide) double staining (Figure 2C), Western blot analysis of cleaved PARP (poly ADP-ribose polymerase) and cleaved caspase3 (Figure 2D), and Hoechst staining of apoptotic bodies (Figure 2E). Similarly, silencing COX2 with siRNA could also sensitize DCA to kill cervical cancer cells (Supplementary Figure 4B and 4C). However, silencing COX1 using siRNA had no sensitizing effect to DCA (Supplementary Figure 4D). These results indicate that COX2 is a novel resistance factor of DCA, and selective inhibition of COX2 sensitizes DCA to induce apoptosis in cervical cancer cells.

### DCA upregulates COX2 via enhancing its mRNA stability

**Figure 3:**
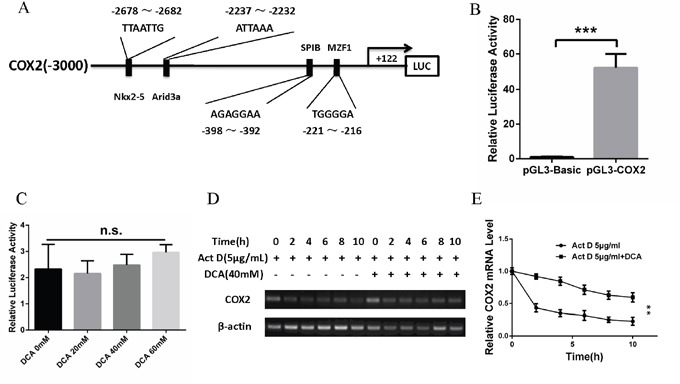
DCA upregulates COX2 via enhancing its mRNA stability **(A)** Schematic representation of *COX2* promoter region containing the putative binding sites for several transcription factors. The region (-3000 to +122) was cloned into pGL3-Basic reporter vector and the resulting plasmid was named pGL3-COX2. **(B)** After transfection with pGL3-COX2 or control vector pGL3-Basic for 24 h, the luciferase activity was assayed using the Dual-Luciferase Reporter System. **(C)** After transfection with pGL3-COX2 for 12 h, HeLa cells were treated with the indicated doses of DCA for 24 h. Then the luciferase activity was assayed using the Dual-Luciferase Reporter System. **(D, E)** HeLa cells were treated with 5 μg/mL actinomycin D (Act D) in the presence or absence of 40mM of DCA for the indicated times, then the level of COX2 mRNA was assayed by PCR (D) and qPCR (E). n.s.: no significance; **p<0.01; ***p<0.001.

To explore how DCA upregulates COX2, we first detected whether DCA promoted the transcription of COX2. As shown in Figure 3A, the promoter region of *COX2* gene (-3000 to +122) containing several predicted transcription factor binding sites was successfully cloned into pGL3-Basic reporter vector, and the resulting plasmid was named pGL3-COX2. Reporter assay showed that the luciferase activity of pGL3-COX2 was significantly higher than that of pGL3-Basic (Figure 3B). Moreover, DCA had no significant influence on the luciferase activity of pGL3-COX2 (Figure 3C), suggesting that DCA may not affect the transcriptional activity of *COX2* gene promoter. Next, we examined the effect of DCA on the mRNA stability of COX2 in cervical cancer cells. As shown in Figure 3D and 3E, cotreatment with DCA and the transcription inhibitor actinomycin D (Act D) decreased the level of COX2 mRNA more slowly than the treatment with Act D alone, indicating that DCA upregulates COX2 through increasing its mRNA stability.

### DCA increases COX2 mRNA stability via downregulating QKI (quaking)

**Figure 4:**
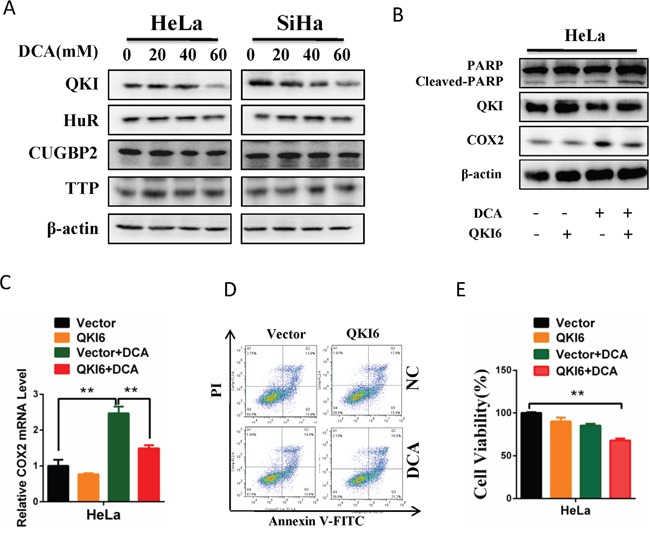
DCA increases COX2 mRNA stability through downregulating QKI **(A)** HeLa and SiHa cells were treated with the indicated doses of DCA for 24 h, and then the expression of RNA binding proteins including QKI, HuR, CUGBP2 and TTP were detected by Western blot. **(B, C)** After transfection with pcDNA3.1-QKI6 or control vector pcDNA3.1 for 12 h, HeLa cells were treated with 40 mM DCA for 24 h. Then the protein levels of cleaved PARP, QKI and COX2 were examined by Western blot (B), and the mRNA level of COX2 was measured by qPCR (C). **(D, E)** HeLa cells were treated as in (B), then the percentage of apoptotic cells was calculated using flow cytometry after stained with annexin V-FITC/PI (D), and the cell viability was detected by CCK8 assay (E). QKI6: pcDNA3.1-QKI6; Vector: control vector pcDNA3.1; **p<0.01.

The stability of mRNA can be critically regulated by RNA binding proteins. To investigate the underlying mechanism by which DCA increases COX2 mRNA stability in cervical cancer cells, we measured the changes of 4 RNA binding proteins which may regulate COX2 via a post-transcriptional way after treatment with different concentrations of DCA. As shown in Figure 4A, DCA markedly reduced the protein level of QKI but had no effect on HuR (Hu antigen R), CUGBP 2(CUG triplet repeat RNA-binding protein 2) and TTP (tristetraprolin) in cervical cancer cells. Moreover, overexpression of QKI dramatically attenuated DCA-induced COX2 and significantly strengthened apoptosis in the presence of DCA (Figure 4B-4E). Collectively, these data demonstrate that DCA increases COX2 mRNA stability by attenuating QKI.

### Celecoxib enhances the chemosensitivity of cervical cancer cells to DCA *in vivo*

**Figure 5:**
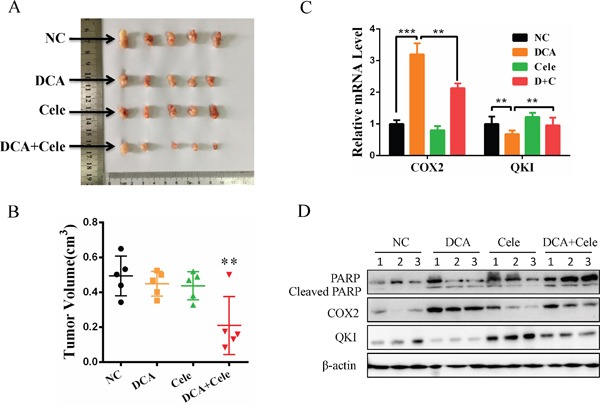
Celecoxib enhances the chemosensitivity of cervical cancer cells to DCA *in vivo* **(A-D)** Each nude mouse was implanted with 5×10^6^ HeLa cells in 150 μL PBS into the right axillae. When the tumors were formed, the mice were randomized into 4 groups (n=5 per group), and then separately treated with PEG, DCA (50 mg/kg/d), celecoxib (30 mg/kg/d) and DCA plus celecoxib every two days. Ten days later, the xenograft tumors were photographed (A) and the tumor volumes were estimated using the following formula: volume = width^2^×length×1/2 (B). The mRNA levels of QKI and COX2 were detected by qPCR (C) and the protein levels of cleaved PARP, QKI and COX2 were examined by Western blot (D). NC: negative control; **p<0.01.

As shown in Figure 5A and 5B, cotreatment with DCA and celecoxib suppressed the growth of cervical cancer xenografts in nude mice more efficiently compared to the treatment with DCA alone. Analogously, DCA increased the mRNA and protein levels of COX2 and decreased the mRNA and protein levels of QKI in the xenograft tumors (Figure 5C). Moreover, the combination of celecoxib and DCA remarkably augmented the cleaved PARP compared to DCA alone in the xenograft tumors (Figure 5D). These results indicate that inhibition of COX2 can enhance the chemosensitivity of DCA in cervical cancer cells *in vivo*.

## DISCUSSION

DCA has been widely used as a single agent or sensitizer in various types of human cancer cells and animal tumor models [11]. DCA can reverse mitochondrial dysfunction and reactivate mitochondria-dependent apoptosis in several tumor cells by inhibiting the activity of PDK, which subsequently promotes the flux of carbohydrates into mitochondria and thereby enhances aerobic oxidation of glucose [8, 10–11, 37–38]. However, in the present study, we found that DCA could suppress the growth of cervical cancer cells only at a high concentration, indicating that DCA is relatively ineffective in cervical cancer cells, unlike it shows in other cancer types. Therefore, it is urgent to explore the resistant determinant factor of DCA in cervical cancer. Previous studies have demonstrated that overexpression of COX2 is strongly correlated with the development and progression of various types of cancers [20, 23]. In this study, we for the first time revealed that DCA induced COX2 *in vitro and in vivo*, and inhibition of COX2 with celecoxib or siCOX2 increased the sensitivity of cervical cancer cells to DCA by promoting apoptosis, indicating that COX2 is a novel resistance factor of DCA in cervical cancer. Indeed, COX2 is upregulated in cervical tumor tissues and predicts a poor prognosis. Moreover, COX2 can be induced by many other chemotherapeutic drugs and reduced their therapeutic effects [30]. For example, COX2 promotes the repopulation of early bladder urothelial carcinomas and decreases cytotoxicity of gemcitabine and cisplatin [39]. The underlying mechanism may be that COX2 is a master regulator of PEG2 production, which contributes to a pro-tumorigenic inflammatory microenvironment and protects tumor from killing by chemotherapeutic drugs. It is well known that pro-tumorigenic microenvironment inflammatory factors (such as IL-6, IL-1β) can promote tumor progression and remarkably impede therapy responses [40]. In our study, IL-1β, IL-6, IL-8 and TNFα were significantly induced by DCA, which were paralleled with the upregulation of COX2. These inflammatory factors may activate downstream signal pathways and finally lead to apoptosis resistance of DCA in cervical cancer cells [41]. Therefore, inhibition of COX2 by celecoxib or siCOX2 may also sensitize other chemotherapeutics which may be compromised by the elevated COX2. Additionally, we also explored the role of COX1 (another subtype of COX family members) in the sensitivity of cervical cancer cells to DCA. The results showed that DCA had no obvious effect on COX1 expression and knockdown of COX1 could not enhance the apoptosis of cervical cancer cells in response to DCA, which suggests that COX1 may be not a resistance factor of DCA.

Although DCA elevated the COX2 mRNA level in cervical cancer cells, the luciferace reporter assay showed that the transcriptional activity of *COX2* gene promoter was not changed by DCA. Interestingly, we found that the half-time of COX2 mRNA increased upon DCA treatment. It has been reported that RNA binding proteins (RBPs) are key regulators of mRNA decay, and some RBPs can regulate COX2 mRNA stability through binding to the ARE ( AU-rich elements) in its 3′-UTR (3′ untranslated region) [42–44]. For instance, HuR can enhance whereas CUGBP2 and TTP can decrease COX2 mRNA stability by binding to its 3′-UTR [44]. However, in the present study, we found that HuR, CUGBP2 and TTP were not involved in the regulation of DCA-induced COX2. QKI (quaking), which belongs to the STAR (signal transduction and activation of RNA) family of KH domain containing RNA binding proteins, is highly conserved across different species [45–46]. Through recognizing the mRNA sequence with special characteristics (NACUAAY-N(1–20)-UAAY), QKI can regulate the location, stability and translational efficiency of target mRNA to modulate physiological and pathological processes [45, 47–48]. Bioinformatics analysis predicts that there are multiple potential quaking response elements (QRE) in the 3'-UTR of COX2 mRNA. Importantly, DCA could downregulate QKI and overexpression of QKI could markedly alleviate the DCA-mediated elevation of COX2 mRNA, suggesting that QKI may play an important role in regulating the stability of COX2 mRNA in the presence of DCA. However, the detailed mechanism(s) by which QKI suppresses the stability of COX2 mRNA remains to be further studied.

In summary, we demonstrated in this study that the DCA-induced COX2 impedes the antitumor effect of DCA in cervical cancer cells, and inhibition of COX2 by celecoxib can sensitize DCA in suppressing the growth of cervical cancer cells, which may pave a way for developing novel strategies for the treatment of cervical cancer using the combination of DCA and celecoxib.

## MATERIALS AND METHODS

### Cell lines and reagents

Human cervical cancer cell lines including HeLa and SiHa were from the American Type Culture Collection (ATCC), and cultured in Dulbecco's Modifed Eagle Medium (DMEM), supplemented with 10% fetal bovine serum (FBS), streptomycin (100 mg/mL) and penicillin (100U/mL) at 37°C in a 5% CO_2_ humid incubator. DCA and celecoxib were purchased from Sigma-Aldrich (Louis, MO, USA) and Selleck (Shanghai, China) respectively. Hoechst 33258 was from Beyotime Company (Shanghai, China). Annexin V-FITC and PI were bought from BD Bioscience (BD, NJ, USA). pcDNA3.1 and pcDNA3.1-QKI6 were gifted from Professor Zifan Lu, Fourth Military Medical University, Xi’an, China.

### Cell viability assay and RwT-CES analysis

The cell viability was assayed using a CCK-8 kit (Dojindo, Shanghai, China). Briefly, the cells were seeded into 96-well plates and given different treatments in triplicate for 24 h, and then 10 μL CCK-8 solution was added to each well. After incubation at 37°C for 1.5 h, the value of OD_450nm_ was determined with a microplate reader. For the RT-CES analysis using ACER xCELLigence System, 50 μL culture media was added to each well of the cell culture E-plates purchased from ACER Biosciences Inc (Hangzhou, China). After measuring the baseline signals, cervical cancer cells were seeded into each well at a density of 10,000 cells per well and incubated 24 h at 37°C and 5% CO_2_. Then the cells were treated with different agents and the impendence of each well was recorded in a 15-min interval for 96 h, and the kinetic curve of cell growth was plotted.

### Western blot

Cells were harvested and the whole-cell lysates were prepared. Then Western blot was performed as previously described [49]. The antibodies for COX2, PARP and cleaved caspase3 were from Cell Signaling Technology (Boston, MA, USA). The antibodies for COX1 and QKI were from Novus Biologicals (Littleton, CO, USA). The antibody for β-actin was from Abcam Company (San Francisco, CA, USA). The antibodies for HuR, CUGBP2 and TTP were from Proteintech Group (Wuhan, China).

### Flow cytometry

Cervical cancer cells were harvested and incubated with annexin V-FITC and PI according to the manufacturer's instructions (Bio-Rad, Shanghai, China). Then the apoptosis were analyzed by a flow cytometer.

### Hoechst staining

After treated for 24 h, the cells were stained with Hoechst 33258 (Beyotime, Shanghai, China) at 10 μg/mL for 10 min in the dark at room temperature. Then the cells were washed 3 times with PBS and photographed under a fluorescence microscope.

### Transfection assay

After grown to 70%–80% confluence, the cells were transfected with the plasmid or siRNA using Lipofectamine 2000 according to the manufacturer's instruction. After 6 h, the transfection medium was replaced by DMEM with 10% FBS, and cultured for another 6 h. Then the cells were given the corresponding treatment.

### Luciferase reporter assay

HeLa cells were seeded into 48-well plates and grown to 70%–80% confluence. Then the cells were cotransfected with pGL3-COX2 (GeneCopoeia, Guangzhou, China) and monitor plasmid pRL-TK (Promega, Madison, USA). After 12 h, the cells were given different treatment for 24 h. Then the Firefly and Renilla luciferase activities were measured using the Dual-Luciferase Reporter System (Promega, Madison, USA) according to the manufacturer's instructions.

### RNA isolation and quantitative real-time polymerase chain reaction (qPCR)

Total RNA was extracted from the cells with TRIzol reagent (ComWin Biotechnology, Beijing, China) as described previously [50], and the first-strand cDNA was synthesized using PrimeScriptTM RT reagent Kit (Takara, Dalian, China). Then qPCR was performed with SYBR qPCR master mix (ABI, NY, USA) according to the manufacturer's instruction.

### Animal study

Six-week-old female nude mice were purchased from Beijing Vital River Experimental Animal Co. Ltd. (China), and housed and cared for under the regulations of the guidelines of the Animal Care and Ethics Committee of Third Military Medical University (Chongqing, China). Each mouse was implanted with 5×10^6^ HeLa cells in 150 μL PBS into the right axillae. When the tumors formed, the mice were randomized into 4 groups (n=5 per group), and then treated with control, DCA (50 mg/kg/d), celecoxib (30 mg/kg/d) and DCA plus celecoxib every two days. Ten days later, the xenograft tumor size was monitored with sliding caliper, and the tumor volume was estimated using the following formula: volume = width^2^×length×1/2. After excised from the mice, the xenograft tumors were photographed, and the corresponding proteins and mRNAs were examined by Western blot and qPCR, respectively.

### Statistical analysis

Data were presented as means±standard deviation (SD). Statistical significances were evaluated by One-way ANOVA and *t*-test. *P*<0.05 was considered as statistical significant.

## SUPPLEMENTARY MATERIALS AND FIGURES


