# Supplementary material for: Inhibition of COX2 enhances the chemosensitivity of dichloroacetate in cervical cancer cells
Source: Oncotarget. 2017 Jun 16;8(31):51748–57. doi: 10.18632/oncotarget.18518 (PMC5584284; doi:10.18632/oncotarget.18518)
Supplement: Supplementary file 1 [file oncotarget-08-51748-s001.pdf]

Inhibition of COX2 enhances the chemosensitivity of dichloroacetate in cervical cancer cells

SUPPLEMENTARY MATERIALS

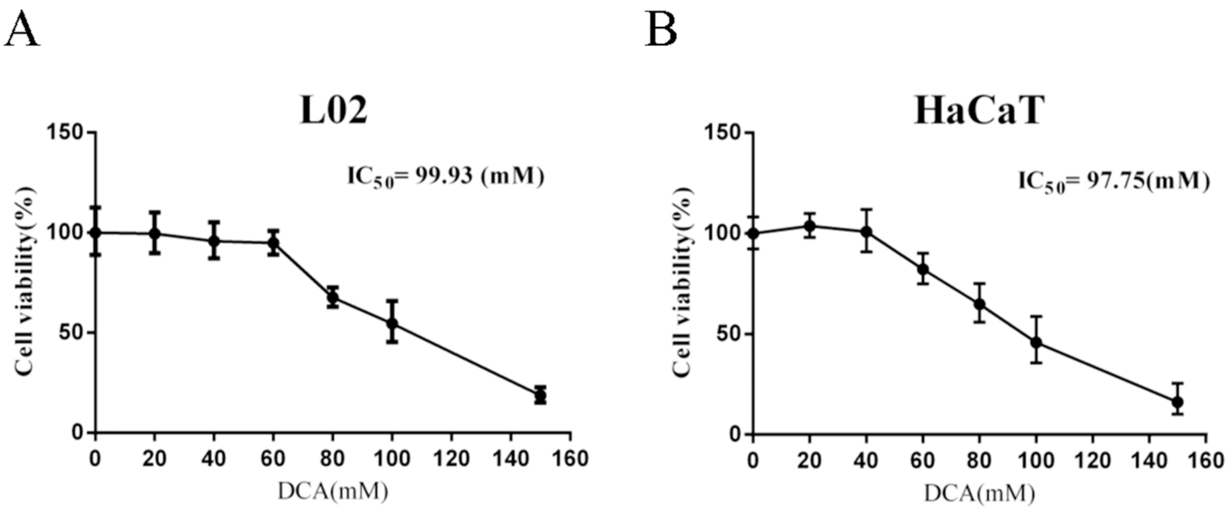

Supplementary Figure 1: DCA has no effect on the growth of L02 and HaCaT cells. (A, B) L02 and HaCaT cells were seeded into 96-well plates and treated in triplicate with different doses of DCA for 24 h, and then the CCK8 assay was performed to evaluate the cytotoxicity of DCA.

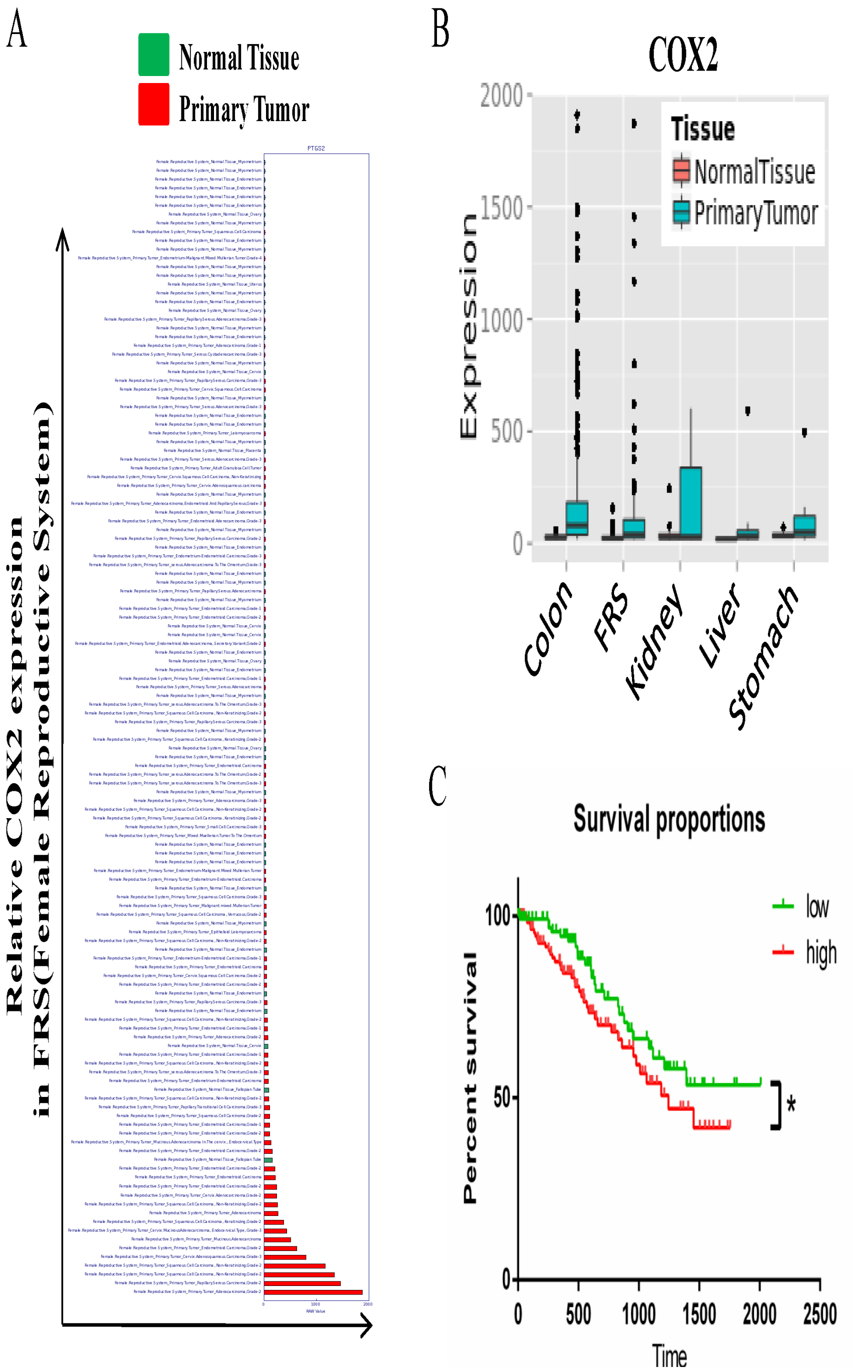

Supplementary Figure 2: Bioinformatics analysis indicates that COX2 is upregulated in tumor tissues which results in a poor prognosis in cervical cancer. **(A)** Expression of COX2 in normal tissues and primary tumors of FRS (female reproductive system). COX2 was subjected to a search in MERAV for the expression in FRS. The results represent the bar graph (generated by MERAV). The bars colors were manipulated (a feature in the MERAV) in order to indicate the tissues of origin. The color legend was shown in the upper corner. **(B)** Expression of COX2 in different normal tissues. The same search parameters as in **(A)**, with the results presented as a boxplot. This figure was generated using MERAV without any additional tools. **(C)** According to an analysis of the *TCGA_CESC_exp_HiSeqV2-2015-02-24* dataset, the overall survival curve was plotted and the Cox regression analysis was used to evaluate the prognostic value of COX2 in cervical tumor tissues. *p<0.05.

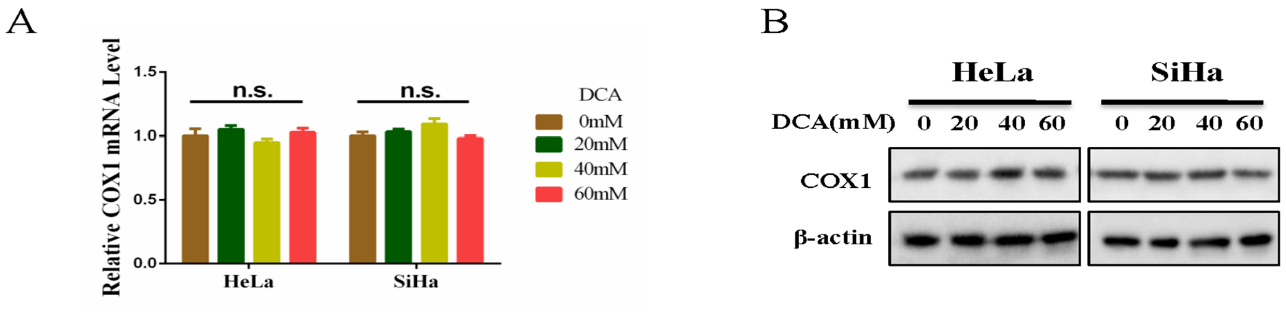

Supplementary Figure 3: DCA has no effect on the expression of COX1. **(A, B)** HeLa and SiHa cells were treated with the indicated doses of DCA for 24 h, then the levels of COX1 mRNA and protein were separately examined by qPCR **(A)** and Western blot **(B)**. n.s.: no significance.

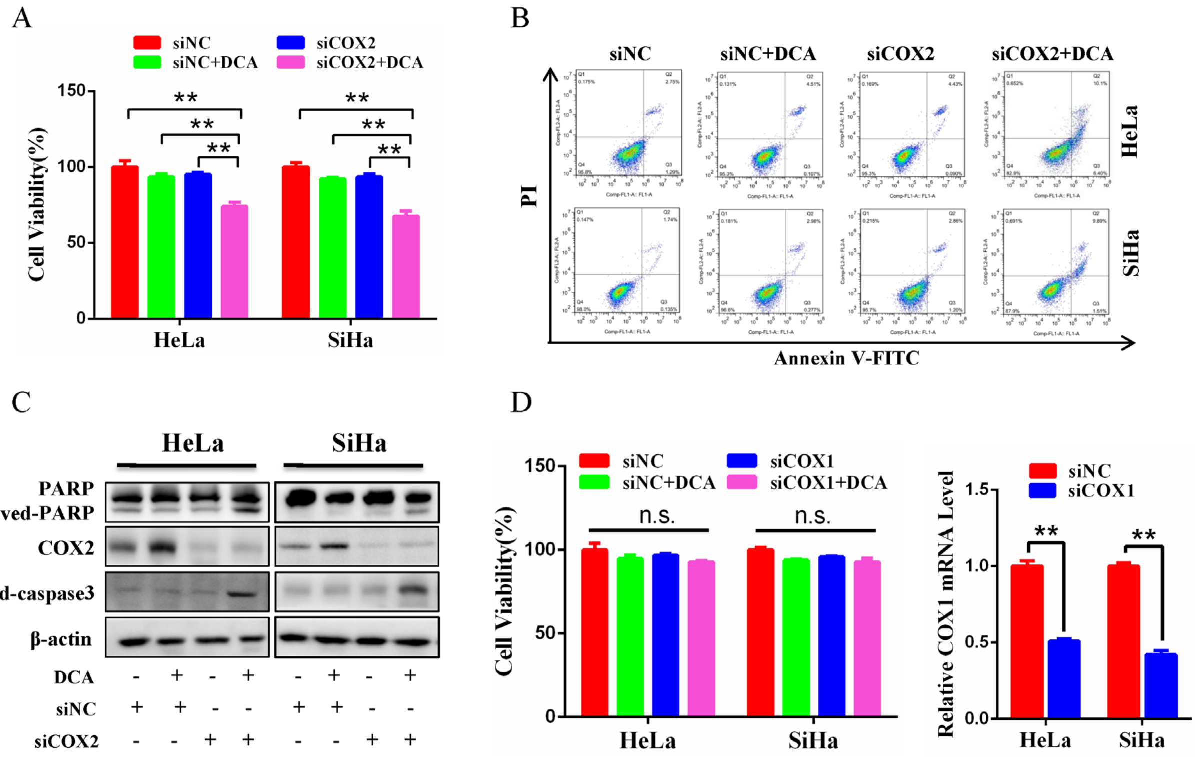

Supplementary Figure 4: Inhibition of COX2 by siRNA sensitizes DCA in cervical cancer cells. **(A-C)** After transfection with COX2 siRNA or control siRNA for 12 h, HeLa and SiHa cells were treated with 40 mM DCA or PBS control for 24 h. Then the cell viability was detected using CCK8 assay **(A)**, the percentage of apoptotic cells was calculated using flow cytometry after stained with annexin V-FITC/PI **(B)**, and the protein levels of COX2, cleaved PARP and cleaved caspase3 were examined with Western blot **(C)**. **(D)** HeLa and SiHa were treated as in **(A)**, and then the cell viability was detected using CCK8 assay and the level of COX1 mRNA was examined by qPCR. siNC: siRNA for negative control; siCOX2: siRNA for COX2; siCOX1: siRNA for COX1; ***p*<0.01; n.s.: no significance.
